# Characterization and Schematic Modeling of Oxidized Fat Use in Swine Feeding: Metabolic and Productive Consequences—A Review

**DOI:** 10.3390/ani16040578

**Published:** 2026-02-12

**Authors:** Luis Humberto López-Hernández, Gerardo Ordaz-Ochoa, Edwin Giovanni Negrete-Morales, María Alejandra Pérez-Alvarado

**Affiliations:** 1 Centro Nacional de Investigación Disciplinaria en Fisiología y Mejoramiento Animal, Instituto Nacional de Investigaciones Forestales, Agrícolas y Pecuarias (INIFAP), Colón, Querétaro 76280, Mexico; lopez.lhumberto@inifap.gob.mx (L.H.L.-H.); perez.maria@inifap.gob.mx (M.A.P.-A.); 2 Facultad de Estudios Superiores en Medicina Veterinaria y Zootecnia, Universidad Nacional Autónoma de México, Cuautitlán 54740, Mexico; giovannineg@comunidad.unam.mx

**Keywords:** oxidized fats, swine nutrition, energy metabolism, oxidative stress, meat quality

## Abstract

Lipid sources are essential components in modern swine nutrition, not only due to their high energy density but also because of their positive effects on palatability, feed efficiency, and micronutrient absorption. However, rising raw material costs have encouraged the use of oxidized fats and oils (OxFO) as a cost-effective alternative in pig diets. These lipids, degraded by thermal and handling factors, undergo chemical alterations that negatively affect digestibility, energy metabolism, and animal health. This review critically examines the current scientific evidence regarding the impact of oxidized fat consumption in swine production systems. The physiological and biochemical mechanisms by which lipid oxidation products impair mitochondrial β-oxidation, cellular oxidative balance, energy efficiency, and meat quality are discussed. Moreover, the practical consequences on productive performance, muscle oxidative stability, and the expression of inflammatory and antioxidant markers are explored. Findings suggest that although the use of oxidized fats may offer economic savings, their metabolic and productive repercussions can compromise profitability and sustainability. The need to define safe inclusion thresholds (when replacement is not feasible), standardize analytical methods to assess oxidation status, and consider nutritional alternatives to mitigate adverse effects is emphasized.

## Simple Summary

Dietary fats are commonly used in pig nutrition to increase energy intake and feed efficiency. However, the use of oxidized fats has become more frequent due to economic constraints. Oxidation products formed during fat deterioration can impair digestion, energy metabolism, and oxidative balance, leading to reduced growth efficiency and poorer meat quality. This review summarizes current evidence on the metabolic and physiological effects of oxidized fats in pigs and highlights the importance of controlling lipid quality and implementing nutritional strategies to minimize their negative impact on swine production.

## 1. Introduction

Lipid sources represent one of the most energy-dense components in swine diets [1,2]. Beyond their caloric contribution, dietary fats improve palatability, reduce feed dustiness, and enhance the absorption of fat-soluble vitamins, collectively promoting feed efficiency and growth performance [3]. Consequently, the proper selection and handling of dietary lipid sources is critical to ensuring productive and economic performance in intensive pig production systems [4,5].

However, the sustained increase in the cost of conventional feed ingredients has prompted the industry to consider more economical alternatives. Among these, recycled or low-quality fats and oils—often exhibiting varying degrees of oxidation—have gained attention [6,7]. Oxidized lipids are the result of chemical degradation processes induced by heat, light, oxygen, and pro-oxidant metals, leading to the formation of hydroperoxides, aldehydes, ketones, and polar compounds that compromise nutritional value and safety [4,8]. In this review, the term oxidized fats and oils (OxFO) refers specifically to dietary fats and oils of triacylglycerol origin that have undergone oxidative degradation during processing, storage, or handling. This definition excludes other lipid classes encompassed by the broader term “lipids,” such as phospholipids, glycolipids, sterols, and related compounds. This distinction is essential, as OxFO represent the primary lipid fraction used as energy sources in swine diets and the main contributors to oxidative challenges associated with feed lipid deterioration.

The inclusion of OxFO in swine diets has been associated with adverse effects on multiple physiological and productive parameters. Beyond productive performance, a significant adverse effect of OxFO is their detrimental impact on animal health. Experimental evidence indicates that the consumption of oxidized fats and oils can impair intestinal integrity, disrupt gut barrier function, and promote inflammatory responses, thereby compromising nutrient absorption [9,10,11,12]. In addition, systemic exposure to lipid oxidation products has been associated with oxidative damage in metabolically active organs, particularly the liver and intestine, contributing to organ dysfunction and reduced physiological resilience [13,14].

Despite the growing body of research documenting the negative impacts of OxFO, the current literature lacks a systemic and integrative framework to assess their effects holistically. In this context, the application of theoretical models such as the General Systems Theory becomes relevant to analyze the interplay between animal, technological, and environmental components within production systems [15,16]. This theory suggests that biological systems—such as intensively reared pigs—should be evaluated considering the dynamic interactions between their internal components and the surrounding environment, particularly when the system is challenged by inputs of variable quality.

This review aims to provide a critical and schematic overview of the effects of OxFO in swine feeding, with a focus on the physiological, biochemical, and technological changes associated with lipid peroxidation, lipid digestion and metabolism, animal energy efficiency, and meat quality. The goal is to offer an integrated perspective to guide future research and practical decision-making regarding the use of recycled lipids, within a framework of sustainability, metabolic efficiency, and animal health. The increasing use of alternative lipid sources in animal feeds highlights the need for comprehensive evaluation of fat quality, as emphasized in recent integrative reviews addressing OxFO in practical feeding systems [2].

## 2. Methodological Approach

For the characterization and schematic modeling of the use of oxidized fats and oils (OxFO) in swine feeding and their relationship with energy metabolism, scientific literature was reviewed using major academic databases, including Web of Science, Scopus, PubMed, ScienceDirect, and Google Scholar. The search covered publications from January 2000 to December 2024 and was conducted between January and March 2025.

Search terms were selected to capture studies related to oxidized fats and oils in swine nutrition and their metabolic, physiological, and productive effects. The main keywords included combinations of oxidized fats, oxidized oils, lipid oxidation, peroxidized lipids, swine, pigs, pig nutrition, energy metabolism, oxidative stress, intestinal health, mitochondrial function, and meat quality. Boolean operators were applied as appropriate (e.g., “oxidized fats AND pigs”, “lipid oxidation AND swine nutrition”, “oxidized oils AND meat quality”).

Studies were considered eligible if they met the following inclusion criteria: (i) peer-reviewed articles published in English; (ii) experimental or observational studies conducted in pigs at any production stage; and (iii) studies evaluating dietary oxidized fats and oils or lipid oxidation products with outcomes related to energy utilization, metabolic responses, oxidative stress, intestinal integrity, organ function, or meat quality. Review articles were also included when they provided relevant mechanistic or conceptual insights. Exclusion criteria included studies conducted in non-porcine species, articles focusing exclusively on non-dietary lipid oxidation (e.g., postmortem oxidation only), and reports lacking clear methodological descriptions or relevance to animal nutrition. Titles and abstracts were initially screened for relevance, followed by full-text evaluation of selected articles. Additional references were identified through manual screening of reference lists from key publications.

The collected information was subsequently analyzed through the lens of General Systems Theory (GST), a theoretical framework that supports the integration of multiple scientific disciplines to address complex problems in a holistic manner [15]. In real-world scenarios, processes and phenomena often defy classification within a single discipline due to their inherent complexity, resulting in what are known as complex systems [16,17].

Swine production systems can be conceptualized as complex systems composed of interactions among four key elements: context, human input, the animal itself, and technology [16]. In this review, emphasis was placed on the technological component, as it is a critical determinant of operational efficiency. This component manifests at two levels: a physical level, encompassing tools and processes used to reduce system variability (e.g., lipid source selection), and a biological level, involving applied knowledge to regulate physiological functions such as lipid digestion and metabolism [18].

Considering these dimensions, the impact of OxFO in swine feeding was examined through two analytical modeling approaches. The first was a general or “black box” approach, in which both endogenous and exogenous factors influencing energy metabolism are assessed without decomposing the system into its constituent parts. The second was a formal or “open box” model, allowing for a more precise exploration of internal relationships. This approach integrates concepts such as functional homogeneity, interdependence among components, cross-disciplinary knowledge, and evaluation under specific hypotheses [19].

To represent the metabolic changes induced by OxFO consumption, conceptual diagrams were used as analytical tools. These schematic representations simplify and visualize key interactions within the biological system, omitting structural details that are not essential for understanding the phenomenon. Rather than reproducing the system in its entirety, the goal is to functionally represent observable or inferable relationships that are otherwise too complex to be analyzed collectively. This approach is consistent with the principles of GST, which emphasize that modeling aims not to mimic reality but to partially reconstruct it through representations that facilitate scientific analysis [20].

## 3. Factors Attributable and Non-Attributable to the Biological Component (Pig) That Modify Energy Metabolism When Consuming Oxidized Fat Sources

### 3.1. Context

The use of feed-grade fats and oils, including rendered fats, recycled lipid streams, and refining co-products, is a common practice in modern swine production systems, largely driven by the sustained increase in the cost of conventional feed ingredients and the need to optimize dietary energy density [6,7,21]. These lipid sources are frequently characterized by heterogeneous chemical composition and variable oxidative status as a consequence of processing intensity, repeated thermal exposure, storage duration, and handling conditions [6,7,8,9,10,22].

Evidence summarized in the present review indicates that a considerable proportion of commercially available fats and oils used in pig diets contain measurable concentrations of primary and secondary lipid oxidation products, reflecting inconsistent quality control across production chains and supply routes [9,10,11,12,23]. This variability is of particular relevance because pigs are highly sensitive to changes in dietary lipid quality, and exposure to oxidized fats and oils has been associated with alterations in energy utilization efficiency, oxidative status, intestinal integrity, and metabolic regulation [9,10,11,12,24].

From a productive and economic perspective, experimental studies cited in this article report that the inclusion of oxidized fat sources in swine diets can result in reductions in average daily gain, feed intake, and feed efficiency, alongside increased oxidative stress and metabolic disturbances, effects that ultimately translate into diminished productive efficiency under commercial conditions [11,12,13,14,25]. Collectively, these findings support the notion that the presence of oxidized fats and oils in practical feeding systems represents a non-negligible risk factor for swine productivity and metabolic health, justifying the need for a systems-based evaluation of their impact on energy metabolism.

### 3.2. Human Component

Within the framework of GST, the human component represents the decision-making and knowledge-driven element that actively regulates the functioning of the production system. In the context of OxFO use in swine feeding, this component encompasses formulation strategies, ingredient selection, quality control, interpretation of analytical indicators, and the implementation of nutritional or technological interventions aimed at mitigating metabolic disturbances. Although pigs are among the most extensively studied livestock species, the physiological consequences of OxFO consumption—particularly those related to energy metabolism, oxidative stress, and meat quality—are often addressed in a fragmented manner [12,24,26]. This fragmentation limits the ability of nutritionists and production managers to establish objective thresholds, safety margins, and corrective strategies when degraded lipids are incorporated into diets.

**Figure 1:**
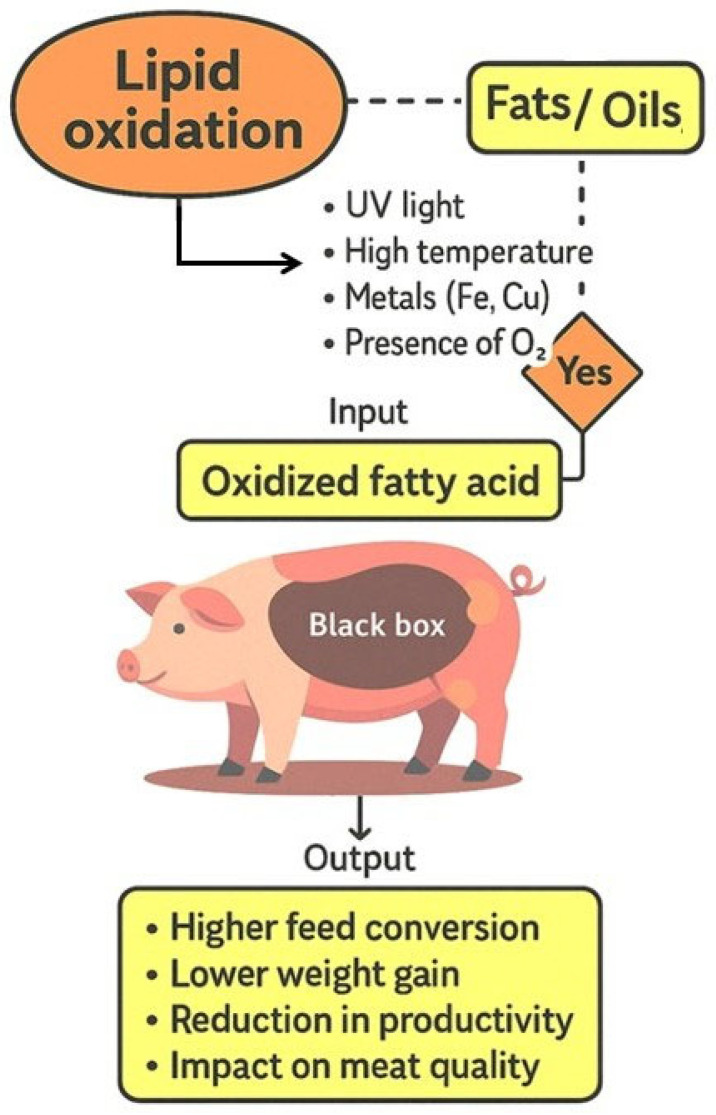
Schematic “black box” representation of the inputs and outputs of the biological system associated with the consumption of oxidized fats and oils.

From a systems perspective, the human factor does not merely act as an external operator but as an integrative regulator that links economic pressures, technological capabilities, and biological responses [27]. Decisions such as accepting oxidized lipid sources based solely on cost, selecting inadequate analytical indicators, or overlooking cumulative oxidative load can propagate negative effects throughout the system, ultimately reducing productive efficiency and product quality. Therefore, strengthening the human component through system-based interpretation, standardized lipid quality assessment, and evidence-driven nutritional strategies is essential for controlling the metabolic and productive consequences associated with OxFO inclusion in swine diets (Figure 1).

### 3.3. Animal Component

Animal production systems are artificial constructs in which the animal depends entirely on human intervention to meet its physiological needs [28]. Thus, the greater the extent to which the biological component [the animal] is supported within the system, the greater the productive output. However, achieving this requires not merely the physical presence of the animal in the system, but a deep understanding of its biology [15]

**Figure 2:**
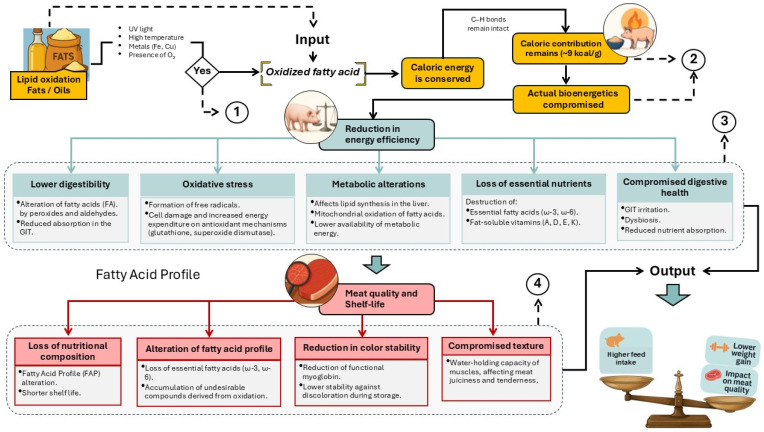
Structured diagram of the porcine biological system integrating physiological, technological, and contextual variables associated with the consumption of oxidized lipids. Numbers 1, 2, 3, and 4 serve as visual connectors referring to complementary sub-diagrams, where the involved functional mechanisms are described in greater detail.

Addressing the challenges and physiological effects associated with the inclusion of OxFO in swine feeding necessitates a shift from a relatively simple system—composed of human, animal, and technology—to a more complex configuration: the modulation of energy metabolism in pigs in response to oxidized lipid intake (Figure 2). Starting from this analytical premise, and by advancing our understanding of this phenomenon, it becomes feasible to regulate and strategically control the use of OxFO in swine nutrition.

### 3.4. Technological Component

From the perspective of GST, the technological component in the study of OxFO in swine nutrition refers to the set of tools, processes, and scientific applications used to analyze, control, and mitigate the negative effects of these lipids on animal metabolism and product quality. This component integrates transversally across multiple critical subtopics within the pig production system [29,30]. The implications of lipid processing, handling, and quality control for energy estimation and nutritional value in animal feeds have been comprehensively discussed by [2].

In addition to analytical and nutritional tools, technological processes involved in lipid production, processing, handling, and storage play a central role in determining the oxidative status and energetic value of fats and oils used in swine diets [31]. Industrial operations such as rendering, refining, cracking, repeated thermal exposure, and prolonged storage under inadequate conditions accelerate lipid oxidation by promoting the formation of hydroperoxides and secondary oxidation products. Moreover, exposure to oxygen, light, transition metals, and moisture during transport and on-farm handling further exacerbates lipid deterioration, chemically altering fat structure and increasing oxidative susceptibility. These technological factors can dilute the effective metabolizable energy of lipids through the accumulation of moisture, impurities, unsaponifiable compounds (e.g., waxes and complex lipids), and non-elutable material [32]. Such dilutive components have been shown to reduce lipid energy content by up to 46% [31], highlighting the risk of overestimating both energy contribution and lipid quality when re-purposing fats originally intended for human consumption in swine feeding systems [33]. As lipid oxidation progresses, an increase in fatty acid saturation occurs, which plays a central role in the negative effects of OxFO on energy metabolism in pigs [5,12].

In the analysis of lipid peroxidation, technology is reflected in the use of advanced analytical methods to quantify oxidative damage. In the domain of energy utilization and efficiency, technologies such as indirect calorimetry and mitochondrial biomarkers are employed to assess the metabolic impact of oxidized lipids. Likewise, in the study of lipid digestion and metabolism, digestive technologies—including exogenous enzymes and emulsifiers—are applied to enhance the absorption of degraded fats. Finally, in meat quality evaluation, the technological component includes instrumental tools for the analysis of color, volatile compound profiles, and texture, as well as nutritional strategies to preserve muscle oxidative stability. Collectively, these technological applications are essential for understanding and intervening in swine production systems that incorporate OxFO into the diet, supporting evidence-based decision-making and precision nutrition practices.

## 4. Lipid Peroxidation

### 4.1. Major Drivers and Causes of Lipid Oxidation

**Figure 3:**
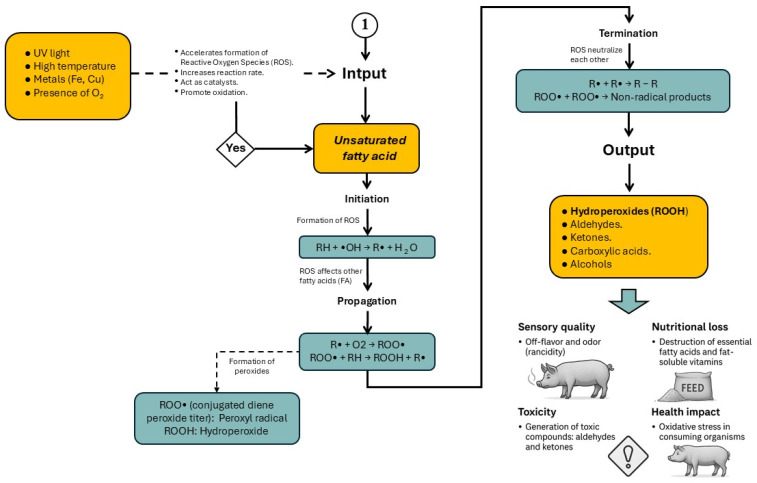
Phases and key compounds generated during lipid peroxidation: initiation, propagation, and termination. Modified from Belitz et al. [29].

The peroxidation of fats and oils is a complex process influenced by several factors, including the degree of fatty acid saturation, temperature, and the presence of oxygen, transition metals (e.g., Cu and Fe), undissociated salts, water, and various non-lipid compounds [4]. Lipid peroxidation proceeds through three main stages: initiation, propagation, and termination (Figure 3), with each phase both consuming and generating a variety of chemical compounds [29].

During the early stages of peroxidation, lipid hydroperoxides are formed, which not only degrade lipid quality but also promote the generation of secondary and tertiary oxidation products. These include aldehydes, ketones, alcohols, hydrocarbons, volatile organic acids, and epoxide compounds, all of which can exert detrimental effects on animal productivity and health [4,8]. The accumulation of these oxidative by-products in feed lipids reduces palatability, disrupts nutrient digestibility, and contributes to oxidative stress at the cellular level.

A comprehensive overview of the sources, characterization, and biological implications of OxFO in animal feeds has been provided by Shurson et al. [2], highlighting the complexity of lipid oxidation processes and the need for integrated analytical and nutritional approaches when evaluating lipid quality in practical feeding systems.

### 4.2. Mechanisms and Biological Consequences of Lipid Peroxidation

The inclusion of oxidized fats in animal diets can negatively impact feed palatability, nutrient digestibility, and overall performance [5,26]. In pigs, consumption of diets containing OxFO has been consistently associated with reduced feed efficiency and lower weight gain [12].

Beyond performance parameters, lipid peroxidation compromises the nutritional quality of feed ingredients by reducing the bioavailability of fat-soluble vitamins and essential antioxidants [12]. Oxidative by-products can also interact with other biomolecules, such as proteins and nucleic acids, altering their structure and function and contributing to the development of pathological conditions [8,29]. Notably, dietary exposure to OxFO has been shown to induce oxidative stress in tissues, a condition linked to cellular damage and impaired reproductive function in animals [12,31].

### 4.3. Detection and Quantification of Lipid Oxidation Products

Due to the complexity of lipid peroxidation and the wide array of compounds formed and degraded throughout the process, accurately quantifying the oxidative status of feed lipids remains a significant analytical challenge [30]. Consequently, no single method can adequately characterize or predict the degree of lipid oxidation [4]. Various analytical techniques are therefore employed, including spectrophotometric and chromatographic methods, each with distinct advantages and limitations depending on the sample matrix, target compounds, and available analytical resources.

In practical feed manufacturing and quality control, exhaustive characterization of all oxidation products is rarely feasible. Instead, routine evaluation relies on a limited number of analytical indicators that collectively reflect different stages of lipid oxidation. Among these, peroxide value (PV) is the most widely used parameter to estimate primary oxidation products, whereas p-anisidine value (p-AV) and thiobarbituric acid reactive substances (TBARS) are commonly applied to quantify secondary oxidation compounds, particularly aldehydes and malondialdehyde [4,30,34]. In addition, methods such as the active oxygen method (AOM) and the oxidative stability index (OSI) are frequently used to assess the resistance of lipids to further oxidation during processing and storage [33,35,36].

### 4.4. Interpretation and Limitations of Oxidation Indicators

Although universally accepted regulatory limits for lipid oxidation in animal feed are lacking, the literature indicates that lipid sources with PV below approximately 5–10 mEq O_2_/kg and relatively low p-AV or TBARS values are generally considered acceptable for use in swine diets, particularly when inclusion levels are moderate and antioxidant protection is adequate [4,33]. However, these reference ranges must be interpreted cautiously, as oxidation kinetics, fatty acid composition, processing history, and antioxidant depletion can markedly influence the biological relevance of a given analytical value [2].

Importantly, no single parameter is sufficient to fully characterize the oxidative status of a lipid source. Peroxide value may underestimate oxidation at advanced stages due to hydroperoxide decomposition, whereas secondary oxidation indicators fail to capture early oxidative changes [4,30]. Likewise, stability tests provide predictive information rather than a direct measure of existing oxidative damage [33,36]. Consequently, a combined assessment integrating at least one indicator of primary oxidation (e.g., PV), one of secondary oxidation (e.g., p-AV or TBARS), and one measure of oxidative stability (e.g., OSI or AOM) is recommended as a practical and biologically meaningful approach for evaluating fats and oils used in swine feeding systems [4,33].

## 5. Energy Utilization in Pigs Fed Oxidized Fats

Energy efficiency in pig production is a critical determinant of performance and profitability. Fats and oils serve as concentrated sources of energy in swine diets; however, they are rarely evaluated at the biological level due to the complexity, cost, and labor-intensive nature of the procedures involved. Such evaluations often require controlled experimental conditions and the use of live animals, making them impractical for routine quality control in feed manufacturing [4,32].

Lipid sources used in swine nutrition vary widely in their physicochemical characteristics—including color, fatty acid profile, free fatty acid content, degree of saturation/unsaturation, saponification value, and impurities [4,5]. Regarding the oxidative status of fats used in pig diets, a broad range of oxidation levels has been reported across different lipid sources (Table 1) [33].

**Figure 4:**
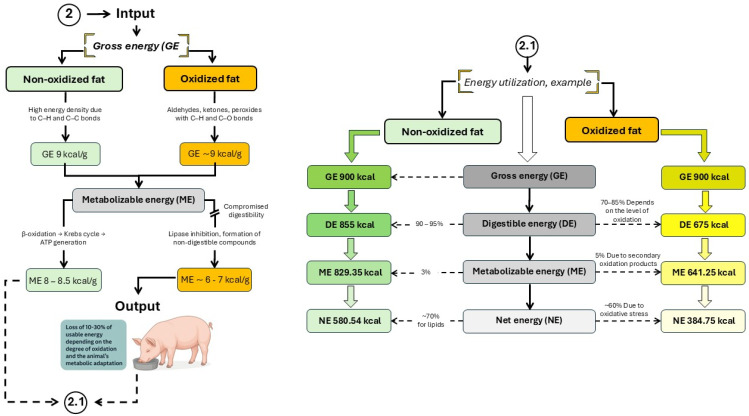
Energy utilization in pigs consuming oxidized fats and oils. Developed based on the findings reported by Kil et al. [3]; Li et al. [9]; Lindblom et al. [11] and Liu et al. [12].

Although the number of studies specifically addressing the use of oxidized fats in swine nutrition is still limited, available evidence indicates that diets containing OxFO have detrimental effects on pig growth performance [5]. These effects include reduced weight gain, slower growth rates, increased systemic oxidative stress, and lower energy digestibility [4,9,12,26]. The reduction in productive performance observed in pigs fed OxFO-rich diets has been closely linked to impaired energy utilization mechanisms (Figure 4).

The amount of usable energy a pig can derive from dietary lipids depends on species, age, and the chemical composition of the fat source [34]. For lipids, metabolizable energy typically accounts for 98–99% of digestible energy, and net energy represents approximately 88–90% of metabolizable energy [9,35]. However, accurate prediction of fat utilization is complicated by endogenous fat losses (EFL). In growing pigs, reported EFL values range from 1.33 to 14.02 g/kg of dry matter intake, reflecting variability driven by the type and amount of fat in the diet [34]. Diets rich in unsaturated fatty acids have been shown to increase fecal fat excretion due to microbial hydrogenation of unsaturated fatty acids in the hindgut [1,4].

Consequently, both overestimation and underestimation of dietary lipid energy content are common. This variability results from differences not only among lipid sources but also within the same source, especially when the energy value has not been empirically determined [2]. For instance, net energy values of choice white grease measured experimentally differed by up to 14% compared to estimates based on NRC (2012) equations [34]. To improve estimation accuracy, it has been proposed to incorporate detailed fatty acid profiles into predictive models [1], as natural variation in lipid composition can significantly impact energy availability—particularly in fats and oils derived from the food industry.

## 6. Consumption of Oxidized Fats and Oils and Their Relationship with Energy Efficiency in Pigs

**Figure 5:**
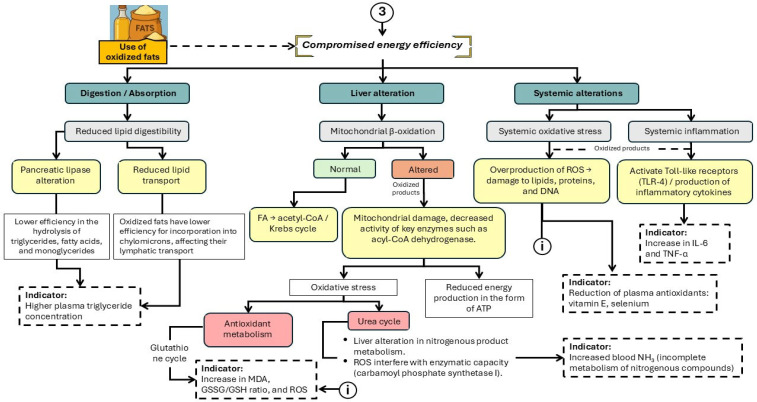
Main physiological alterations associated with the consumption of oxidized fats and oils that interfere with energy efficiency in pigs. FA: fatty acids; ROS: reactive oxygen species; IL-6: Interleukin-6; TNF-α: Tumor necrosis factor-alpha; MDA: Malondialdehyde; GSSG/GSH: oxidized-to-reduced glutathione ratio.

The use of OxFO in swine nutrition poses an emerging risk to both energy efficiency and metabolic health. While lipids serve as a concentrated energy source in diets for growing pigs, oxidation—caused by inadequate processing, storage, or reuse—leads to the formation of reactive compounds that may disrupt multiple metabolic pathways [37] (Figure 5).

The schematic representation summarizes the main physiological disruptions induced by OxFO consumption. These include reduced lipid digestibility and mitochondrial damage, which impair β-oxidation and ATP production [38]. In addition, systemic effects such as oxidative stress, inflammation mediated by Toll-like receptor 4, and alterations in the urea and glutathione cycles have been observed. These disruptions compromise overall energy efficiency and are associated with elevated plasma markers of cellular damage, including malondialdehyde (MDA), the glutathione redox ratio, interleukin-6 (IL-6), and tumor necrosis factor-alpha (TNF-α) [39,40,41].

### 6.1. Lipid Digestion and Metabolism

Under normal physiological conditions, lipid digestion in pigs begins in the oral cavity, where salivary enzymes such as lingual lipase and esterases contribute to the initial hydrolysis of triglycerides. This activity is particularly important in neonatal piglets [42], although its relevance decreases with age [43]. Mastication facilitates the release and subsequent emulsification of dietary lipids. In the stomach, triglycerides are partially converted into diglycerides and free fatty acids, which can trigger hormonal responses such as the release of cholecystokinin, a key regulator of intestinal motility and satiety [44,45]. However, the contribution of gastric lipases in pigs is limited, as they are inactivated under acidic pH conditions [43,46].

The principal site of lipid digestion is the small intestine, where more than 70% of dietary fats still reach the lumen in triglyceride form [47]. Non-emulsified fats are initially coated with bile salts and phospholipids, allowing the action of pancreatic enzymes including lipase, colipase, and cholesterol esterase [48,49]. Colipase stabilizes lipase at the lipid–water interface, facilitating triglyceride hydrolysis into monoglycerides and free fatty acids [50]. These products are incorporated into mixed micelles for transport to the enterocytes, where they are absorbed either by passive diffusion (short- and medium-chain fatty acids) or via active transport mechanisms (long-chain fatty acids) [50,51]

However, the inclusion of OxFO in the diet can significantly disrupt this process. At the digestive level, OxFO may impair pancreatic lipase activity and reduce the efficiency of lipid emulsification and transport, particularly by altering the incorporation of triglycerides into chylomicrons [52]. These disruptions result in reduced lipid absorption, increased circulating triglycerides, and lower energy availability. In addition, OxFO can impair mixed micelle formation, a key process in lipid digestion whereby fatty acids and monoacylglycerols associate with bile salts and phospholipids to form soluble complexes that facilitate lipid transport across the unstirred water layer of the intestinal lumen [9]. The presence of lipid oxidation products alters the physicochemical properties of these micelles, reducing lipid solubilization and intestinal uptake [11]. As a consequence, diminished micellar transport limits the availability of absorbed fatty acids for re-esterification within enterocytes, indirectly disrupting *de novo* lipid synthesis and chylomicron assembly, thereby altering lipid absorption efficiency and postabsorptive lipid metabolism [24,53].

In post-absorptive metabolism, oxidized fatty acids may induce mitochondrial dysfunction in the liver, especially by impairing β-oxidation through reduced activity of key enzymes such as acyl-CoA dehydrogenase, thereby compromising ATP production. Moreover, oxidative lipid by-products generate reactive oxygen species (ROS), triggering systemic oxidative stress, intestinal inflammation, and activation of immune pathways such as toll-like receptor 4, leading to the release of proinflammatory cytokines including IL-6 and TNF-α [54,55]. These alterations not only reduce the energy efficiency of the diet but also impair intestinal health, cellular membrane integrity, and the bioavailability of essential nutrients such as vitamin E and selenium, both of which are reduced in animals fed oxidized lipids [55].

### 6.2. Mitochondrial β-Oxidation

Under normal physiological conditions, β-oxidation is the primary catabolic pathway for the energy utilization of fatty acids in pigs. This process takes place within the mitochondrial matrix and begins with the transport of long-chain fatty acids into the mitochondria via the carnitine–carnitine palmitoyltransferase I system, located on the outer mitochondrial membrane [56]. Once inside, fatty acids undergo sequential degradation by a set of enzymes—acyl-CoA dehydrogenase, enoyl-CoA hydratase, 3-hydroxyacyl-CoA dehydrogenase, and 3-ketoacyl-CoA thiolase—producing acetyl-CoA, NADH, and FADH_2_. These products feed into the Krebs cycle and the electron transport chain to support ATP synthesis [57].

However, when pigs consume OxFO, this process is impaired. Lipid metabolism inherently produces ROS, which disrupt mitochondrial function and increase oxidative stress, thereby reducing energy efficiency [11,58]. Excess ROS damage mitochondrial membranes and oxidative machinery, leading to reduced ATP production and negatively impacting growth and feed conversion efficiency [59].

In pigs fed OxFO, significant reductions in the NADH/NAD^+^ ratio and in cytochrome c oxidase activity have been reported, indicating compromised metabolic coupling and decreased respiratory chain efficiency [60,61]. At the systemic level, elevated plasma acylcarnitine concentrations have been observed, reflecting an accumulation of incompletely oxidized fatty acid intermediates and mitochondrial β-oxidation dysfunction [59].

From an enzymatic standpoint, chronic consumption of oxidized lipids impairs the activity of key enzymes such as long-chain acyl-CoA dehydrogenase and carnitine palmitoyltransferase I, with activity reductions of up to 40% reported [62,63]. This inhibition limits fatty acid entry into mitochondria and delays their catabolism, resulting in the accumulation of free fatty acids and increased hydrogen peroxide production, a toxic by-product that exacerbates oxidative damage in hepatic and muscular tissues [64].

The metabolic consequence of this imbalance is lipotoxicity, characterized by the accumulation of intermediates such as diacylglycerols and ceramides that interfere with cellular energy homeostasis [65]. In pigs, this condition is reflected in elevated plasma concentrations of non-esterified fatty acids [66]. This increase in non-esterified fatty acids is not only a marker of lipid mobilization but is also associated with hepatic dysfunction, insulin resistance, and accelerated protein catabolism. These findings demonstrate that the consumption of OxFO compromises mitochondrial β-oxidation, not only reducing the pig’s ability to extract energy from lipids but also promoting a pro-oxidative environment that impairs cellular health, growth, and overall production efficiency.

## 7. Consumption of Oxidized Fats and Its Relationship with Meat Quality

**Figure 6:**
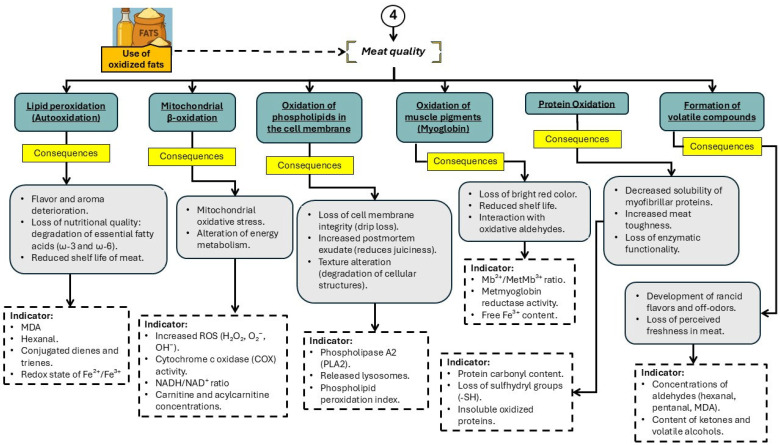
Main physiological alterations associated with the consumption of oxidized fats and oils that affect meat quality in pigs. ROS: reactive oxygen species; MDA: Malondialdehyde.

As previously discussed, the use of OxFO in swine diets is a common practice in the pork industry due to their low cost and availability [27]. However, beyond adversely affecting animal metabolism, growth, and health, the inclusion of OxFO also has detrimental consequences on the final quality of pork [67,68,69]. This impact is primarily linked to the disruption of several key metabolic pathways, including lipid peroxidation, mitochondrial β-oxidation, phospholipid oxidation in cell membranes, myoglobin oxidation, protein oxidation, and the generation of volatile compounds (Figure 6). These alterations compromise important meat quality traits such as oxidative stability, color, tenderness, water-holding capacity, and sensory attributes. The accumulation of oxidative products in muscle tissue, including MDA and protein carbonyls, has been associated with lipid rancidity, discoloration, and increased drip loss—factors that directly reduce consumer acceptance and shelf life of pork products.

### 7.1. Lipid Peroxidation and Oxidative Stress

The consumption of oxidized fats introduces lipid hydroperoxides and free radicals into the pig’s organism, promoting oxidative stress and disrupting cellular homeostasis [70]. Lipid peroxidation involves the reaction of polyunsaturated fatty acids with molecular oxygen, generating highly reactive secondary products such as MDA and 4-hydroxynonenal, which can trigger cell damage and programmed cell death (apoptosis) in various tissues [71,72]. These peroxidation products compromise the integrity of cellular membranes, increasing membrane permeability and impairing cellular function—factors that directly impact meat stability.

Diets enriched with oxidized fats have been shown to increase MDA levels in plasma and muscle tissues (Table 2), with values ranging from 2.5 to 4.0 nmol/g of fresh meat. In contrast, pigs fed fresh lipids exhibit MDA levels below 2.0 nmol/g [14,71]. Likewise, hexanal—a volatile compound derived from n-6 fatty acid peroxidation—has been detected at concentrations exceeding 1.2 µg/g in deteriorated pork (Table 2), contributing to rancid flavors and reduced consumer acceptance [13].

Oxidative stress induced by lipid peroxidation also disrupts the activity of endogenous antioxidant defense systems. In pigs fed oxidized lipids, the activity of key enzymes such as superoxide dismutase and catalase is reduced (Table 2), compromising the neutralization of ROS [31]. Furthermore, the concentration of reduced glutathione—a key cellular antioxidant—can decrease by up to 40%, enhancing the propagation of oxidative damage in muscle tissues and accelerating *postmortem* degradation of meat [73].

Another critical consequence of lipid peroxidation is its impact on inflammation and immune response. Ingested oxidized lipids activate pro-inflammatory signaling pathways such as nuclear factor kappa B, leading to elevated production of cytokines including IL-6 and TNF-α [74]. In pigs exposed to high-OxFO diets, plasma IL-6 levels can increase up to 2.5-fold compared to pigs fed fresh lipids, suggesting a chronic inflammatory state that may impair growth and overall welfare.

The deterioration of oxidative stability due to lipid peroxidation not only shortens meat shelf life but also compromises its sensory and nutritional attributes [75]. Lipid oxidation in skeletal muscle results in the degradation of essential fatty acids and the loss of fat-soluble vitamins, such as vitamins E and A, thereby reducing the nutritional value of the final product [76].

### 7.2. Oxidation of Phospholipids in Cellular Membranes

One of the less visible but critically harmful effects of feeding pigs oxidized lipid sources is the structural and functional damage to cellular membranes. These membranes, composed largely of phospholipids containing polyunsaturated fatty acids, are particularly susceptible to lipid peroxidation in the presence of free radicals and ROS [75,77]. Oxidation of these lipids compromises not only the physical integrity of the lipid bilayer but also essential functions such as ion transport, intracellular signaling, and transmembrane receptor function [78]. In monogastric animals such as pigs, chronic exposure to oxidized lipids has been associated with a significant increase in phospholipid hydroperoxide levels, destabilizing both plasma and mitochondrial membranes [5,33].

This structural damage triggers a cascade of functional alterations. Loss of membrane integrity facilitates the release of lysosomal enzymes and activates apoptotic pathways, undermining cell viability and ultimately affecting the quality of muscle tissue [79]. Mitochondrial membranes, in particular, are highly vulnerable to this process, leading to decreased electron transport efficiency and increased ROS production—factors that perpetuate oxidative damage in tissues [80].

Furthermore, phospholipid oxidation alters membrane fluidity, negatively affecting the function of critical transmembrane proteins such as insulin receptors and glucose transporters, with significant metabolic implications [81]. In vivo studies have also reported reduced activity of Na^+^/K^+^-ATPase, a key enzyme for maintaining membrane potential. Impaired function of this enzyme compromises ionic homeostasis and contributes to cellular deterioration in animals exposed to oxidized lipids [82]. In this context, phospholipid oxidation represents a critical link in the chain of events leading to the deterioration of meat quality in pigs fed degraded fats.

### 7.3. Myoglobin Oxidation, Protein Oxidation, and Formation of Volatile Compounds

The oxidative stability of meat is a major determinant of its sensory, functional, and nutritional quality [83]. In this context, the oxidation of key components such as myoglobin, structural proteins, and lipids leads to a cascade of degradative changes that directly affect the color, flavor, texture, and shelf life of meat products [83]. In pigs fed oxidized fats, these processes are intensified by increased levels of ROS and secondary lipid peroxidation products, which exacerbate *postmortem* oxidative deterioration of muscle tissue [4,11,12].

*Myoglobin oxidation:* Myoglobin is a heme protein that serves as an oxygen reservoir and transporter in muscle and is the principal determinant of meat color. Its redox state defines the visual appearance of the product: oxymyoglobin imparts a bright red hue, whereas metmyoglobin (MetMb) confers an undesirable brown coloration [84]. Under oxidative stress—especially in the presence of elevated ROS and reactive aldehydes from lipid peroxidation—the conversion of MbO_2_ to MetMb is accelerated [85]. This conversion not only impairs the appearance of the product but also reflects loss of freshness and advanced oxidation. In pigs fed oxidized fats, MetMb levels have been reported to increase by up to 40% compared to pigs fed fresh lipids, significantly compromising visual quality [86]. Furthermore, this process is associated with a reduced muscle reducing capacity, which is reflected in decreased metmyoglobin reductase activity—an enzyme that normally facilitates the regeneration of the desirable red color in meat [85].

*Protein oxidation:* Skeletal muscle proteins play critical structural and functional roles, including in muscle contraction, nutrient transport, and water retention [87]. Oxidation of these proteins—induced by dietary oxidized lipids—leads to irreversible modifications such as side-chain carbonylation, cross-link formation, and protein fragmentation [88]. These alterations negatively impact texture, reduce myofibrillar protein solubility, and impair water-holding capacity. In swine studies, elevated protein carbonyl content and significant losses of sulfhydryl groups have been observed, both of which are indicators of severe structural damage [89]. Additionally, protein oxidation interferes with *postmortem* enzymatic activity of calpains and cathepsins, disrupting controlled proteolysis during aging and thereby reducing final meat tenderness [90].

*Formation of volatile compounds:* Oxidative degradation of lipids and proteins generates a wide array of volatile compounds that negatively affect the aroma and flavor of meat. These include aldehydes (e.g., hexanal, pentanal, heptanal), ketones, alcohols, and organic acids—by-products of polyunsaturated fatty acid and amino acid oxidation [13,85]. These volatiles are responsible for the development of rancid, metallic, and off-flavor notes, which lower product acceptability. Hexanal concentrations in meat from pigs fed OxFO have been reported to be up to three times higher than those from pigs fed fresh fats [91]. Moreover, these volatiles can interact with proteins and muscle pigments, further exacerbating oxidative deterioration. The typical profile of desirable volatile compounds, such as short-chain alcohols and lactones, is replaced by secondary aldehydes that contribute to sensory rejection [92].

**Table 2:** Oxidative stress biomarkers in tissues potentially affecting meat quality in pigs fed fresh vs. oxidized lipids.

Biomarker	Tissue	Lipid Source		Effect on Meat	Reference
		Fresh	Oxidized		
MDA	Meat, nmol/g	<0.5	>2.0	Accelerates rancidity and reduces oxidative stability.	Boler et al. [31]
	Plasma, nmol/mL	9.15	12.51		Degroote et al. [93]
Hexanal	Meat, µg/g	<0.5	>1.2	Contributes to the development of rancid flavors.	Fernando et al. [91]
SOD	Liver, % reduction	-	10–20	Decreases the antioxidant capacity of the muscle, promoting lipid oxidation.	Lindblom et al. [11]
	Erythrocytes, U/mg	3.2	3.0		Keller et al. [94]
Catalase	Erythrocytes, U/g	94	64	Reduces protection against peroxides, increasing oxidative instability.	Keller et al. [94]
GPx	Erythrocytes, U/g	97	74	Lower antioxidant capacity in the muscle, accelerating meat deterioration.	Keller et al. [94]
	Plasma, U/L	307	334		Degroote et al. [93]
FRAP	Plasma, µmolFe/L	135	144	Indicator of antioxidant capacity; lower values are associated with reduced oxidative stability of meat.	Degroote et al. [93]
IL-6	Plasma, pg/mL	43	85	Associated with inflammatory processes that affect muscle quality.	Silva-Guillen et al. [74]
TNF-α	Plasma, pg/mL	23	199	May induce proteolysis and affect meat texture.	Silva-Guillen et al. [74]

MDA: Malondialdehyde; SOD: Superoxide dismutase; FRAP: Ferric reducing antioxidant power; IL-6: Interleukin-6; TNF-α: Tumor necrosis factor-alpha; GPx: glutathione peroxidase.

Together, the oxidation of myoglobin, proteins, and lipids—and the resulting formation of volatile compounds—represent synergistic mechanisms of meat deterioration in pigs fed oxidized lipids [95]. Incorporating natural or synthetic antioxidants into the diet is a key strategy for mitigating these effects and preserving the sensory and functional quality of the final product.

## 8. General Considerations

While the use of OxFO in swine diets may offer a cost-effective strategy for reducing formulation expenses, it carries significant implications for productive efficiency, energy metabolism, animal health, and meat quality. Scientific evidence consistently shows that lipid oxidation products profoundly disrupt key digestive and metabolic processes, including lipid digestion, intestinal absorption, mitochondrial β-oxidation, and cellular redox balance, ultimately compromising the animal’s physiological homeostasis.

From a production standpoint, the inclusion of oxidized lipids has been associated with reduced feed efficiency, slower growth rates, impaired immune response, and decreased oxidative stability of tissues. These alterations can translate into lower overall economic returns, despite the lower initial cost of the lipid source. Additionally, the final product quality is adversely affected by phenomena such as myoglobin oxidation, formation of undesirable volatile compounds, and oxidative damage to phospholipids and structural proteins—impairing the sensory, technological, and nutritional attributes of pork.

Despite these risks, the use of OxFO remains common in certain production systems, particularly those facing economic constraints or lacking quality control capabilities. Therefore, it is crucial to promote improved lipid ingredient monitoring through reliable analytical methods and to establish science-based inclusion limits. Furthermore, the adoption of antioxidant additives, protective dietary formulations, and targeted nutritional strategies is essential to mitigate the adverse effects of oxidative stress induced by degraded lipids. Collectively, these considerations highlight the need to re-evaluate the use of OxFO in swine nutrition—not merely from an immediate economic standpoint but through a comprehensive lens that integrates metabolic efficiency, animal welfare, and long-term sustainability.

## 9. Future Perspectives

Advancing our understanding of the impact of OxFO in swine nutrition requires integrative approaches that go beyond the assessment of energy metabolism and product quality to encompass the sustainability of the production system. Safe inclusion thresholds must be experimentally established, and reliable mitochondrial and digestive biomarkers should be validated for the early detection of functional impairments. In parallel, it is necessary to optimize the use of rapid analytical technologies to assess lipid oxidation in real time, and to explore mitigation strategies involving antioxidants, emulsifiers, and functional additives. Finally, the integration of omics tools and economic–environmental modeling will allow for more accurate evaluations of the cost–benefit ratio of OxFO use, guiding decision-making towards more efficient, safe, and sustainable production systems.

## Figures and Tables

**Table 1:** Chemical indicators of lipid oxidation in different fat sources subjected to thermal treatments. Modified from Liu et al. [33].

	Soybean Oil ^&^		Corn Oil			Canola Oil			Chicken Fat			Tallow		
	Fresh	Oxidized	OL	SO	RO	OL	SO	RO	OL	SO	RO	OL	SO	RO
PV ^1^, mEq/kg	1	43	1	151	2	1	239	12	1	57	2	1	29	3
p-anisidina ^2^	2	57	<1	61.4	142.9	91	37.0	154.8	3	88	22	4	120	19
TBARS ^3^, μmol/kg	9	149	16	225	119	45	968	622	79	151	58	58	61	41
Hexanal, mg/kg	<1	11	<1	390	83	1	180	59	3	88	22	4	120	19
2,4-decadienal, mg/kg	<1	1.5	72	3728	1345	7	1091	511	30	442	169	47	261	125
HNE ^4^, μmol/kg	<1	1	0	194	594	0	105	221	0	2	0	0	13	6
AOM ^5^, mEq/kg	100	236	103	575	528	112	419	533	4	298	5	<2	6	446
OSI ^6^, h	7.3	<1.0	8.4	<1.0	<1.0	9.2	<1.0	<1.0	24.6	<1.0	<1.0	12.1	<1.0	<1.0

OL = original lipids (lipids were stored as received without antioxidants or heating); SO = slow oxidation (SO lipids were heated for 72 h at 95 °C with constant compressed air flow rate at 12 L/min); RO = lipids (RO lipids were heated for 7 h at 185 °C with constant compressed air flow rate at 12 L/min). ^&^ References: [9,10,11,12,23,24]. ^1^ PV = Peroxide value. ^2^ There is no unit for the p-anisidine value. ^3^ TBARS = Thiobarbituric acid reactive substances. ^4^ HNE = 4-hydroxynonenal. ^5^ AOM = Active oxygen method (measured as PV after 20 h of oxidation). ^6^ OSI = Oxygen stability index (time to exponential increase in conductivity when analyzed at 110 °C).

## Data Availability

All data supporting the findings of this study are available from publicly accessible databases, including PubMed, ScienceDirect, and Google Scholar.
